# Changes in 10-Year Predicted Cardiovascular Disease Risk for a Multiethnic Semirural Population in South East Asia: Prospective Study

**DOI:** 10.2196/55261

**Published:** 2024-09-26

**Authors:** Hamimatunnisa Johar, Chiew Way Ang, Roshidi Ismail, Zaid Kassim, Tin Tin Su

**Affiliations:** 1 South East Asia Community Observatory (SEACO) & Global Public Health Jeffrey Cheah School of Medicine and Health Sciences Monash University Malaysia Subang Jaya Malaysia; 2 Heidelberg Institute of Global Health, Faculty of Medicine University of Heidelberg Heidelberg Germany; 3 Segamat Health Office Ministry of Health Malaysia Segamat Malaysia

**Keywords:** cardiovascular risk trajectory, Framingham risk score, population-based study, low- and middle-income countries

## Abstract

**Background:**

Cardiovascular disease (CVD) risk factors tend to cluster and interact multiplicatively and have been incorporated into risk equations such as the Framingham risk score, which can reasonably predict CVD over short- and long-term periods. Beyond risk factor levels at a single time point, recent evidence demonstrated that risk trajectories are differentially related to CVD risk. However, factors associated with suboptimal control or unstable CVD risk trajectories are not yet established.

**Objective:**

This study aims to examine factors associated with CVD risk trajectories in a semirural, multiethnic community-dwelling population.

**Methods:**

Data on demographic, socioeconomic, lifestyle, mental health, and cardiovascular factors were measured at baseline (2013) and during follow-up (2018) of the South East Asia Community Observatory cohort. The 10-year CVD risk change transition was computed. The trajectory patterns identified were improved; remained unchanged in low, moderate, or high CVD risk clusters; and worsened CVD risk trajectories. Multivariable regression analyses were used to examine the association between risk factors and changes in Framingham risk score and predicted CVD risk trajectory patterns with adjustments for concurrent risk factors.

**Results:**

Of the 6599 multiethnic community-dwelling individuals (n=3954, 59.92% female participants and n=2645, 40.08% male participants; mean age 55.3, SD 10.6 years), CVD risk increased over time in 33.37% (n=2202) of the sample population, while 24.38% (n=1609 remained in the high-risk trajectory pattern, which was reflected by the increased prevalence of all major CVD risk factors over the 5-year follow-up. Meanwhile, sex-specific prevalence data indicate that 21.44% (n=567) of male and 41.35% (n=1635) of female participants experienced an increase in CVD risk. However, a stark sex difference was observed in those remaining in the high CVD risk cluster, with 45.1% (n=1193) male participants and 10.52% (n=416) female participants. Regarding specific CVD risk factors, male participants exhibited a higher percentage increase in the prevalence of hypertension, antihypertensive medication use, smoking, and obesity, while female participants showed a higher prevalence of diabetes. Further regression analyses identified that Malay compared to Chinese (*P*<.001) and Indian (*P*=.04) ethnicity, nonmarried status (*P*<.001), full-time employment (*P*<.001), and depressive symptoms (*P*=.04) were all significantly associated with increased CVD risk scores. In addition, lower educational levels and frequently having meals from outside were significantly associated to higher odds of both worsening and remaining in high CVD risk trajectories.

**Conclusions:**

Sociodemographics and mental health were found to be differently associated with CVD risk trajectories, warranting future research to disentangle the role of psychosocial disparities in CVD. Our findings carry public health implications, suggesting that the rise in major risk factors along with psychosocial disparities could potentially elevate CVD risk among individuals in underserved settings. More prevention efforts that continuously monitor CVD risk and consider changes in risk factors among vulnerable populations should be emphasized.

## Introduction

### Background

Cardiovascular diseases (CVDs) continue to be the main contributor to deaths globally, with a significant proportion of the mortality occurring in low- and middle-income countries (LMICs) rather than in high-income countries [1]. In Malaysia, CVD is the leading cause of death, as it accounts for an estimated 20% of all noncommunicable disease mortality [2], escalating the health care and economic burden over the past decades [3]. As a middle-income country, rapid urbanization with major demographic and socioeconomic changes and concurrent shifts in diet and activity levels contribute to the epidemiological shift toward much higher rates of CVD.

Regular assessments of CVD risk factors, as advocated by the Regional Action Framework for Noncommunicable Disease Prevention and Control in the Western Pacific, can inform lifestyle and medical interventions, potentially preventing cardiovascular-related deaths [4]. Multiple CVD risk factors, by contrast, tend to cluster and interact multiplicatively to promote further vascular risk. As a result, CVD risk factors have been incorporated into risk equations such as the Framingham risk score (FRS), which can reasonably predict CVD over short- and long-term periods [5,6]. Furthermore, as CVD risks may change over time, reassessment of CVD risk is important to identify changes, assess the effectiveness of interventions, and ensure adherence to recommended treatments [7]. Previous large epidemiological European studies analyzing these changes in risk scores have been shown to improve risk stratification beyond the single time point risk score classification [8,9].

Although it has been recognized that adverse levels of risk factors often develop early in life and change over time [10,11], the underlying factors that may influence these changes are not fully understood. Noncommunicable diseases might be preceded by a relatively sudden deterioration in risk factors before disease onset. However, previous research demonstrated that traditional cardiometabolic risk factors do not fully account for or explain the excess burden of CVDs in the population, of which the risk may be additionally explained by mental health factors [12,13]. Moreover, the deterioration in CVD risk factors has also been shown to be amplified by other factors, such as sociodemographic, behavioral, and mental health factors [14]. In LMICs, where research in CVD risk trajectories is still limited, demographic, socioeconomic, behavioral, and mental health factors may be highly prevalent and may contribute significantly to unhealthy CVD risk trajectories. For instance, low socioeconomic conditions are strongly linked to financial stress, psychosocial stress [15], and limited access to health care facilities [16], which could further amplify the deterioration of CVD risk factors such as hypertension and diabetes. Therefore, such insight into these multifaceted factors that interact and influence pathophysiological changes provides indications for optimal preventive actions in diverse global contexts.

While most prior studies have mainly focused on the impact of risk score trajectories on CVD morbidity [9], studies that examine mental health and behavioral factors associated with CVD risk trajectories remain scarce, particularly in LMICs. Factors that may improve CVD risk include higher educational levels [17], stable employment [18], healthy lifestyle behaviors [19] such as balanced diet and regular exercise, and good mental health conditions [20]. Conversely, factors that may lead to deterioration of CVD risk include lower socioeconomic status (SES) [21]; unhealthy behaviors [19] such as poor diet, sedentary lifestyle, smoking, and harmful alcohol consumption; and chronic psychological stress, depression, and anxiety [20]. A better understanding of the underlying factors that may influence worsened CVD risk trajectories would facilitate targeting at-risk population groups.

### Objective

This study aims to examine 5-year changes in CVD risk based on 2 assessments of the FRS and to evaluate concurrent demographic, socioeconomic, mental health, and lifestyle factors that may influence the improvement or deterioration of CVD risk trajectories in a representative sample of the multiethnic semirural adult population in Southeast Asia.

## Methods

### Population and Study Setting

This study used the secondary data stem from the community health survey of the South East Asia Community Observatory (SEACO) in 2013 and 2018. SEACO is a health and demographic surveillance system (HDSS) established in Segamat, Johor state, Malaysia, by Monash University Malaysia in 2011 [22]. SEACO conducted house-to-house interviews to obtain information on demographic and socioeconomic characteristics (eg, age, education, income, and marital status), self-reported health status (eg, hypertension status and diabetes status), and mental health condition from 5 subdistricts in Segamat, particularly, Bekok, Chaah, Gemereh, Jabi, and Sungai Segamat. All individuals aged ≥5 years in the selected 5 subdistricts who participated in the SEACO censuses were invited to complete the survey.

In the community health survey, the respondents aged ≥35 years underwent health screening services (anthropometric measurement, blood pressure [BP], and random glucose) and a personal interview conducted by the trained study personnel.

**Figure 1:**
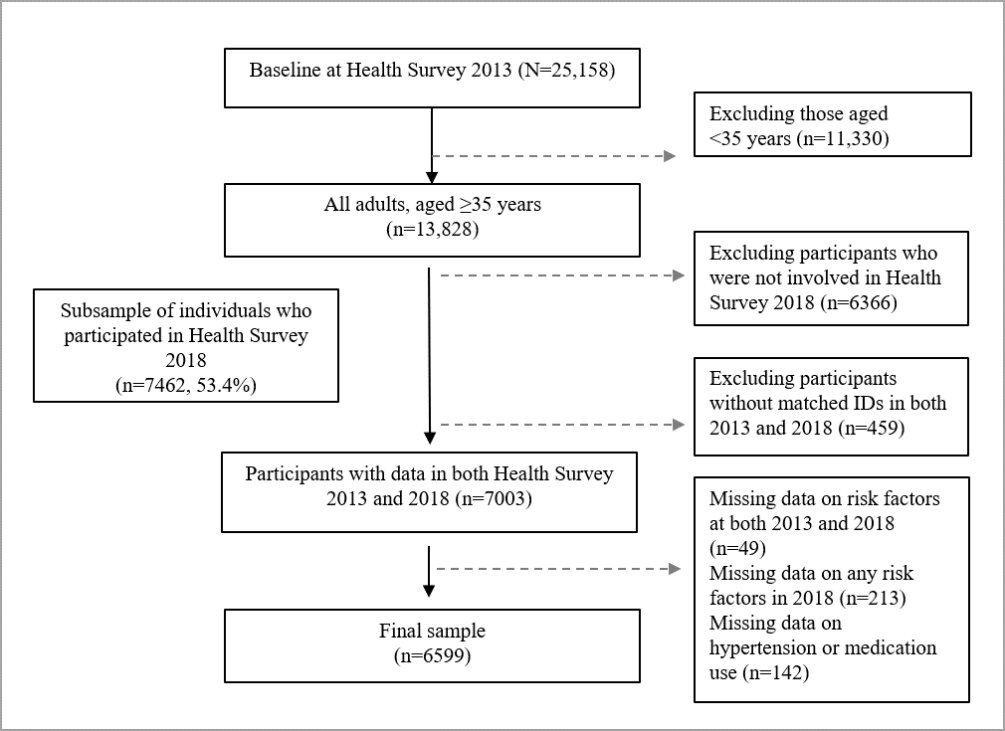
Flowchart of participant selection. At baseline, 13,828 individuals aged ≥35 years were included in the Health Survey 2013 of the South East Asia Community Observatory health and demographic surveillance system, established in Segamat, Johor, Malaysia. A subsample of 7462 individuals was reexamined in the Health Survey 2018. The final study population included 6599 participants who completed both the 2013 and 2018 surveys and provided complete data on cardiovascular disease risk factors.

In 2013, a total of 25,158 individuals aged ≥35 years participated in the Health Survey 2013. Of these, 55% (n=13,828) underwent health screening. A reexamination of the individuals in SEACO HDSS was conducted in 2018. A reexamination of the individuals in SEACO HDSS was conducted in 2018. The SEACO health survey uses a dynamic or open cohort design. However, the study sample was limited to those who participated in both baseline (2013) and follow-up (2018) surveys as well as those without missing data on CVD risk factors at baseline and follow-up, which included 6599 participants, as depicted in Figure 1. In a dropout analysis, excluded participants were more likely to be aged ≥70 years and to have higher FRS scores compared to the study sample (Table S1 in Multimedia Appendix 1). However, no significant sex differences were found between excluded and included participants.

### Outcome Measurement (Cardiovascular Risk Score)

The outcome variable was modified FRS proposed by D’Agostino et al [5] in estimating the 10-year predicted CVD risk among the respondents. The 10-year CVD risk score was calculated using nonlaboratory predictors: age (in years), BMI, use of antihypertensive medication, systolic BP, smoking status, and diabetes mellitus status [5]. Participants who reported “currently smoking” were classified as smokers. Anthropometric measurements, including height (in cm) and weight (in kg), were obtained during home-based interviews. BMI was calculated by dividing weight by height in meters squared. BP was measured 3 times using the Omron HEM 7120 E Blood Pressure Monitor M2 Basic Digital Intellisense, following the standard STEPwise guidelines [23]. For data analysis, the second and third BP readings were averaged, as the first reading may overestimate mean BP [24].

In Malaysia, for primary prevention, the Clinical Practice Guideline committee recommends the use of the FRS general CVD risk score for risk stratification [25]. The FRS score is commonly used as a summary measure in Malaysia and has been shown to predict CVD risk more accurately than other risk scores [26]. Besides that, the modified FRS was used and validated by Su et al [27] in predicting the 10-year CVD risk among the urban population in Malaysia. In this study, the FRS score obtained in 2013 and 2018 were calculated among the study population. The change in CVD risk score over a 5-year period was calculated by subtracting the CVD risk score at follow-up in 2018 from the CVD risk score at baseline in 2013 (CVD risk score at baseline, 2013 – CVD risk score at follow-up, 2018). A negative score indicates an increase in CVD risk (worsened), while a positive score indicates a decrease in CVD risk (improvement).

### Assessment of the CVD Risk Factors and Covariates

The covariates selected were a priori based on the availability of survey questions and the support from the past studies. Several studies showed that there is an association of demographic and socioeconomic characteristics with 10-year CVD risk [28,29], lifestyle practice [30,31], and mental health status [32,33]. The demographic variables included in this study are age, sex, ethnicity, and marital status. Besides that, education, household income, and occupation were included in this study. Due to the lack of extensive dietary data and considering the influence of salt intake on CVD risks among the study population, the frequency of meals taken outside per week was used as the proxy for healthy eating habit, as described previously [31]. Physical activity was assessed using the Global Physical Activity Questionnaire, and data were transformed to derive estimates of the average number of minutes per week engaged in physical activity in 3 domains (occupational, transport, and leisure) and total physical activity at each time point [34]. We classified participants as insufficiently active if they engaged in <600 metabolic equivalent of task minutes of physical activity per week [34]. Symptoms of depression, anxiety, and stress were assessed using the Depression, Anxiety and Stress Scale-21 items [35]. Higher scores indicate greater levels of mental health symptoms. Individuals were classified as having depressive symptoms with a cutoff score of 10 [35].

### Statistical Analysis

Participant characteristics, including demographic, socioeconomic, lifestyle, and mental health factors, are presented for the total sample and according to sex at baseline and follow-up. Categorical variables are summarized as proportions and compared between the 2 time points using chi-square tests, whereas continuous variables are summarized as mean (SD) and compared between the 2 time points were using paired 2-tailed *t* test. Similar analyses were performed to compare the participants’ characteristics and CVD risk groups. To visualize the transition of the predicted 10-year CVD risk change in the study population, we created a river plot using low, moderate, and high-risk CVD groups. To increase the interpretability of the CVD risk trajectories, a stacked bar chart was also created.

Multivariable linear regression models were fit to assess baseline factors associated with changes in CVD risk scores (CVD risk score at baseline, 2013 – CVD risk score at follow-up, 2018). Model 1 was adjusted for demographic and socioeconomic factors (sex, ethnicity, marital status, educational level, income, and employment status). Model 2 was further adjusted for lifestyle factors (frequency of meals taken from outside and physical activity). Model 3 was additionally adjusted for mental health factors (depression, anxiety, and stress). In a sensitivity analysis, multinomial logistic regression models were fitted to assess the association between baseline demographic, socioeconomic, lifestyle, and mental health factors and changes in the predicted CVD risk groups.

We performed complete-case analyses and included participants with available CVD risk factors (at baseline and follow-up) and other covariates data. Descriptive and regression analyses were performed in SPSS (version 20; IBM Corp), and the river plot was created by using the web-based SankeyMATIC software by Steve Bogart [36].

### Ethical Considerations

The surveys were approved by the Monash University Human Research Ethics Committee (MUHREC; MUHREC 3837 [Health Survey 2013, baseline] and MUHREC 13,242 [Health Survey 2018, follow-up]). All participants were given an information sheet and requested to sign a consent form before the data collectors started the interview.

## Results

### Participant Characteristics

A total of 6599 participants (n=3954, 59.92% female participants and n=2645, 40.08% male participants) with a mean age of 55.3 (SD 10.6) years were included in this analysis. Participants’ characteristics of demographic, socioeconomic, lifestyle, clinical, and mental health factors stratified by sex are presented in Table 1. Overall, most participants are categorized in the 50 to 59 years age group (n=2232, 33.83%); of Malay ethnicity (n=4202, 63.68%); were married (n=5551, 84.21%); completed primary school (n=2976, 45.68%); had a monthly household income of <RM 3000 (approximately US $663.35; n=4523, 68.54%); not in full-time employment or self-employment (n=3776, 57.22%); and did not report any symptoms of depression (n=5711, 87.61%), anxiety (n=5425, 82.84%), or stress (n=6253, 95.66%; Table 1). In terms of sex difference, male participants were more likely to be older (men: mean age 56.7, SD 10.7 years; women: mean age 54.4, SD 10.4 years), be married, and currently employed; reported taking more frequent meals from outside; had higher levels of physical activity; and experienced more moderate to severe mental health symptoms than their female counterparts (Table 1). The participants’ baseline demographic (apart from age) and socioeconomic characteristics do not change drastically and are quite stable over the follow-up period (Table S2 in Multimedia Appendix 1).

**Table 1:** Baseline demographic, socioeconomic, lifestyle, and mental health characteristics of a subsample of participants from the South East Asia Community Observatory health survey in 2013 by sex (N=6599).

Variable		Total	Male (n=2645)	Female (n=3954)	Chi-square (*df*)		*P* value^a^	
Age (y), mean (SD)		55.29 (10.60)	56.68 (10.74)	54.36 (10.40)	—^b^		—	
**Age groups (y), n (%)**						14.1 (2)		.001
	35-39	504 (7.63)	175 (6.62)	329 (8.32)				
	40-49	1549 (23.47)	549 (20.75)	1000 (25.29)				
	50-59	2232 (33.83)	808 (30.55)	1424 (36.01)				
	60-69	1665 (25.24)	799 (30.21)	866 (21.90)				
	≥70	649 (9.83)	314 (11.87)	335 (8.48)				
**Ethnicity, n (%)**						5.8 (4)		.21
	Aborigine	74 (1.12)	33 (1.25)	41 (1.04)				
	Chinese	1500 (22.73)	626 (23.67)	874 (22.10)				
	Indian	751 (11.38)	290 (10.96)	461 (11.66)				
	Malay	4202 (63.68)	1674 (63.29)	2528 (63.94)				
	Others	72 (1.09)	22 (0.83)	50 (1.26)				
**Marital status, n (%)**						266.3 (3)		*<*.001
	Married	5551 (84.21)	2411 (91.19)	3140 (79.52)				
	Never married	214 (3.25)	113 (4.28)	101 (2.59)				
	Separated or divorced	115 (1.74)	21 (0.79)	94 (2.37)				
	Widow, widower, or others	712 (10.80)	99 (3.74)	613 (15.52)				
**Education, n (%)**						76.3 (3)		*<*.001
	Primary	2976 (45.68)	1205 (45.85)	1771 (45.57)				
	Secondary	2898 (44.48)	1228 (46.73)	1670 (42.96)				
	Tertiary	236 (3.62)	112 (4.26)	124 (3.19)				
	Others	405 (6.22)	83 (3.16)	322 (8.28)				
**Household income^c^ (RM), n (%)**						9.0 (3)		.03
	<1000	1371 (20.78)	585 (22.12)	786 (19.87)				
	1000-1999	1952 (29.58)	758 (28.66)	1194 (30.20)				
	2000-2999	1200 (18.18)	503 (19.02)	697 (17.63)				
	≥3000	2076 (31.46)	799 (30.20)	1277 (32.30)				
**Occupation, n (%)**						1975.5 (3)		*<*.001
	Paid employee	1577 (23.90)	964 (36.45)	613 (15.50)				
	Self-employed	1246 (18.88)	968 (36.60)	278 (7.03)				
	Not working	724 (10.97)	322 (12.17)	402 (10.17)				
	Others	3052 (46.25)	391 (14.78)	2661 (67.30)				
**Frequency of meals taken outside per week, n (%)**						180.0 (3)		*<*.001
	0	2474 (38.06)	747 (28.79)	1727 (44.21)				
	1-5	2653 (40.81)	1153 (44.43)	1500 (38.40)				
	6-10	643 (9.89)	342 (13.18)	301 (7.71)				
	≥11	731 (11.24)	353 (13.60)	378 (9.68)				
**Level of total physical activity, n (%)**						74.1 (2)		*<*.001
	Low	5830 (88.35)	2256 (85.29)	3574 (90.39)				
	Moderate	365 (5.53)	145 (5.48)	220 (5.56)				
	High	404 (6.12)	244 (9.23)	160 (4.05)				
**Depression, n (%)**						2.7 (3)		.44
	Normal	5711 (87.61)	2264 (86.94)	3447 (88.07)				
	Mild	357 (5.48)	145 (5.57)	212 (5.42)				
	Moderate	340 (5.22)	145 (5.57)	195 (4.98)				
	Severe and extremely severe	110 (1.69)	50 (1.92)	60 (1.53)				
**Anxiety, n (%)**						3.0 (3)		.39
	Normal	5425 (82.84)	2160 (82.32)	3265 (83.18)				
	Mild	276 (4.21)	104 (3.96)	172 (4.38)				
	Moderate	619 (9.45)	260 (9.91)	359 (9.15)				
	Severe and extremely severe	229 (3.50)	100 (3.81)	129 (3.29)				
**Stress, n (%)**						7.5 (3)		.06
	Normal	6253 (95.66)	2494 (95.12)	3759 (96.02)				
	Mild	156 (2.39)	65 (2.48)	91 (2.32)				
	Moderate	88 (1.35)	39 (1.49)	49 (1.25)				
	Severe and extremely severe	40 (0.60)	24 (0.91)	16 (0.41)				

^a^*P* values for differences between male and female participants were calculated using the *χ*^2^ test.

^b^Not applicable.

^c^US $1 is equivalent to RM 3.00 (May 13, 2013).

Table 2 presents the prevalence of CVD risk factors of the study participants at baseline (2013) and follow-up (2018), with all major CVD risk factors showing a significant increase from 2013 to 2018. The prevalence of hypertension increased from 22.47% (n=1483) in 2013 to 38.53% (n=2542) in 2018, as also reflected by the increased antihypertensive medication use (n=980, 14.85% in 2013 to n=1172, 17.76% in 2018). Similarly, an increasing trend was also observed in the prevalence of diabetes (n=795, 12.05% in 2013 to n=1422, 21.55% in 2018), obesity (n=1737, 26.28% in 2013 to n=1871, 28.39% in 2018), and smoking (n=901, 13.65% in 2013 to n=1619, 24.53% in 2018). Similar increasing trends were also observed in systolic BP, BMI, and random blood glucose levels. The mean blood glucose levels increased from 8.0 (SD 3.6) mmol/L in 2013 to 8.3 (SD 4.1) in 2018 (*t*_6597_=–4.7; *P*<.001). Of note, the prevalence of the high CVD risk group significantly increased from 29.78% (n=1965) in 2013 to 44.26% (n=2921) in 2018. Similarly, in comparison to the baseline values, higher mean of total FRS score (2013: mean 11.7, SD 5.5; 2018: mean 14.2, SD 5.4) and overall percentage of predicted CVD risk (2013: mean 14.1%, SD 9.8%; 2018: mean 17.8%, SD 9.9%) at follow-up were also observed (Table S3 in Multimedia Appendix 1).

**Table 2:** Cardiovascular disease risk factors in 2013 and 2018 among the South East Asia Community Observatory study participants (prevalence; N=6599).

Variables		2013, n (%)	2018, n (%)	Chi-square (*df*)		*P* value^a^	
**Hypertension status**					993.7 (1)		*<*.001
	Yes	1483 (22.47)	2542 (38.53)				
	No	5116 (77.53)	4056 (61.47)				
**On antihypertensive medication**					265.7 (1)		*<*.001
	Yes	980 (14.85)	1172 (17.76)				
	No	5619 (85.15)	5427 (82.24)				
**Smoking status**					2408.1 (1)		*<*.001
	Yes	901 (13.65)	1619 (24.53)				
	No	5698 (86.35)	4980 (75.47)				
**Diabetes status**					1392.3 (1)		*<*.001
	Yes	795 (12.05)	1422 (21.55)				
	No	5804 (87.95)	5177 (78.45)				
**BMI**					4900.3 (4)		*<*.001
	Underweight or normal	2327 (35.27)	2128 (32.29)				
	Overweight	2537 (38.45)	2591 (39.32)				
	Obese	1734 (26.28)	1871 (28.39)				
**Predicted cardiovascular risk**					3101.2 (4)		*<*.001
	Low	1820 (27.58)	989 (14.99)				
	Moderate	2814 (42.64)	2689 (40.75)				
	High	1965 (29.78)	2921 (44.26)				

^a^*P* values for differences between baseline (2013) and follow-up (2018) were calculated using the *χ*^2^ test for categorical variables.

The sex-specific prevalence of CVD risk factors in the study population at baseline and follow-up shows an increase in all risk factors from 2013 to 2018 for both male and female participants, as detailed in Table S4 in Multimedia Appendix 1. In 2018, male participants exhibited the highest prevalence of smoking and high predicted CVD risk, while female participants had the highest prevalence of hypertension, diabetes, and obesity. There was a slightly greater increase in the prevalence of diabetes among female participants (2013: n=493, 12.5%; 2018: n=895, 22.6%) compared to male participants (2013: n=302 11.4%; 2018: n=527, 19.9%). This increase in diabetes prevalence was also reflected in a slight increase in blood glucose levels, from 8.1 (SD 4.0) mmol/L to 8.4 (SD 4.1) mmol/L in female participants (*t*_3952_=–3.2; *P*=.02) and from 8.1 (SD 4.0) mmol/L to 8.3 (SD 4.2) mmol/L in male participants (*t*_2644_=–3.3; *P*=.001). Notably, the prevalence of hypertension, antihypertensive medication use, smoking, and obesity showed a higher percentage increase among male participants from 2013 to 2018 compared to female participants (Table 3).

**Table 3:** Sex-stratified cardiovascular disease risk factors in 2013 and 2018 among the South East Asia Community Observatory study participants (prevalence; n=6599).

Variables			Male (n=2645)												Female (n=3954)											
			2013		2018		Chi square (*df*)			*P* value			2013				2018			Chi square (*df*)				*P* value^a^		
**Hypertension status, n (%)**								381.5 (1)			*<*.001											608.8 (1)				*<*.001
	Yes	551 (20.83)		973 (36.80)								932 (23.57)				1569 (39.68)										
	No	2094 (79.17)		1671 (63.20)								3022 (76.43)				2385 (60.32)										
**Antihypertensive medication, n (%)**								259.1 (1)			*<*.001										457.7 (1)				*<*.001	
	Yes	366 (13.84)		488 (18.45)								614 (15.53)				684 (17.30)										
	No	2279 (86.16)		2157 (81.55)								3340 (84.47)				3270 (82.70)										
**Smoking, n (%)**								528.6 (1)			*<*.001										876.1 (1)				*<*.001	
	Yes	874 (33.04)		1577 (59.62)								27 (0.68)				42 (1.06)										
	No	1771 (66.96)		1068 (40.38)								3927 (99.32)				3912 (98.94)										
**Diabetes, n (%)**								533.0 (1)			*<*.001										856.5 (1)				*<*.001	
	Yes	302 (11.42)		527 (19.92)								493 (12.47)				895 (22.64)										
	No	2343 (88.58)		2118 (80.08)								3461 (87.53)				3059 (77.36)										
**BMI**								1826.8 (4)			*<*.001										2986.3 (4)				.001	
	Underweight or normal	1113 (42.08)		1046 (39.55)								1214 (30.71)				1089 (27.54)										
	Overweight	1041 (39.36)		1054 (39.85)								1496 (37.84)				1537 (38.87)										
	Obese	491 (18.56)		545 (20.60)								1243 (31.45)				1328 (33.59)										
**Predicted cardiovascular disease risk**								825.9 (4)			*<*.001										1643.7 (4)				*<*.001	
	Low	194 (7.33)		140 (5.29)								1626 (41.12)				849 (21.47)										
	Moderate	1019 (38.53)		875 (33.08)								1795 (45.40)				1814 (45.88)										
	High	1432 (54.14)		1630 (61.63)								533 (13.48)				1291 (32.65)										

^a^*P* values for differences between baseline (2013) and follow-up (2018) by sex were calculated using the *χ*^2^ test for categorical variables.

With regard to sex differences, female participants had a comparable level of diastolic and systolic BP as well as blood glucose levels to their male counterparts. However, female participants recorded a slightly higher mean of BMI of 28.0 (SD 5.4) compared to male participants, who had a mean BMI of 26.3 (SD 4.5). In terms of high predicted CVD risk, males experienced an increase in prevalence from 54.14% (n=1432) in 2013 to 61.63% (n=1630) in 2018, while female participants showed an increase from 13.48% (n=533) in 2013 to 32.65% (n=1291) in 2018. In addition, male participants exhibited an increase in the mean total FRS score (2013: mean 12.7, SD 4.8; 2018: mean 15.7, SD 5.0), while female participants showed an increase from 11.0 (SD 5.8) in 2013 to 15.1 (SD 9.5) in 2018.

### The Transition of CVD Risk Groups Over Time

**Figure 2:**
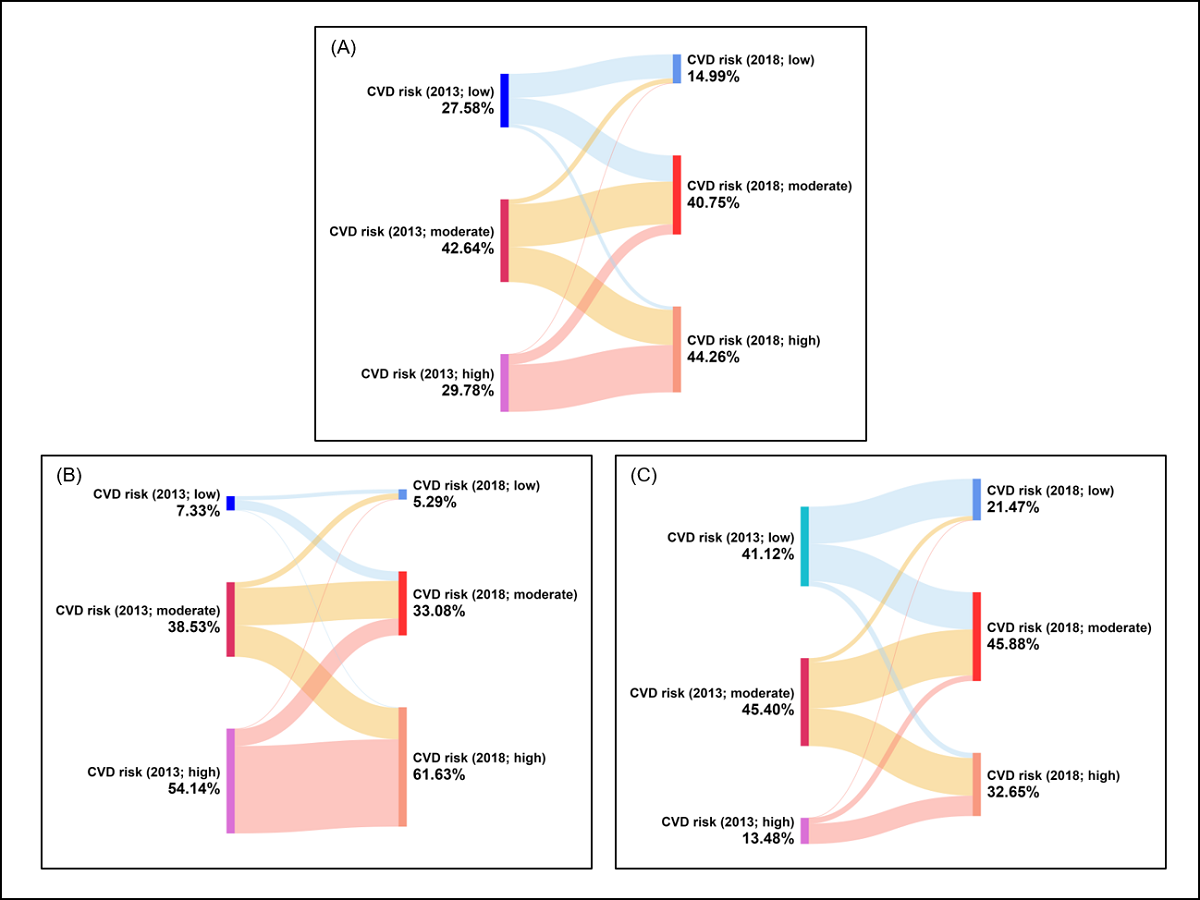
River plot describing transition patterns of low, moderate, or high predicted cardiovascular disease (CVD) risk change from baseline in 2013 to follow-up in 2018 among (A) total population, (B) male population, and (C) female population. Note: The light red lines show the transition of high predicted CVD risk cluster from baseline 2013 branching out to high, moderate or low predicted CVD risk in 2018; the yellow lines indicate the moderate predicted CVD risk cluster from baseline 2013 branching out to high, moderate or low predicted CVD risk in 2018; and the blue lines indicate the low predicted CVD risk cluster from baseline 2013 branching out to high, moderate or low predicted CVD risk in 2018. The percentage (%) values indicate the prevalence of low, moderate, and high CVD risk, based on the 10-year CVD risk classification.

Figure 2 presents river plots that illustrate transitions in CVD risk groups (low, moderate, and high) from baseline in 2013 to follow-up in 2018. On the basis of the 10-year CVD risk classification, the prevalence of low, moderate, and high CVD risk was 27.58% (n=1820), 42.64% (n=2814), and 29.78% (n=1965) at baseline and 14.99% (n=989), 40.75% (n=2689), and 44.26% (n=2921) at follow-up (2018), respectively. The percentage of high predicted CVD risk among the total population had increased significantly from 29.78% (n=1965) in 2013 to 44.26% (n=2921) in 2018, whereas the prevalence of low predicted CVD risk decreased from 27.58% (n=1820) in 2013 to 14.99% (n=989) in 2018. Sex-specific river plots show that male participants were the major contributors to high predicted CVD risk, with a prevalence of 54.14% (n=1432) at baseline in 2013 and 61.63% (n=1630) at follow-up in 2018. In contrast, 13.48% (n=533) of female participants presented with high predicted CVD risk at baseline, which increased to 32.65% (n=1291) at follow-up.

On the basis of the changes from baseline (2013) to follow-up (2018), four distinct trajectory patterns were identified: (1) remained high (n=1609), (2) remained low or moderate (low-low: n=815; moderate-moderate: n=1452), (3) adverse (n=2202), and (4) improved (n=521; Figure S1 in Multimedia Appendix 1).

**Figure 3:**
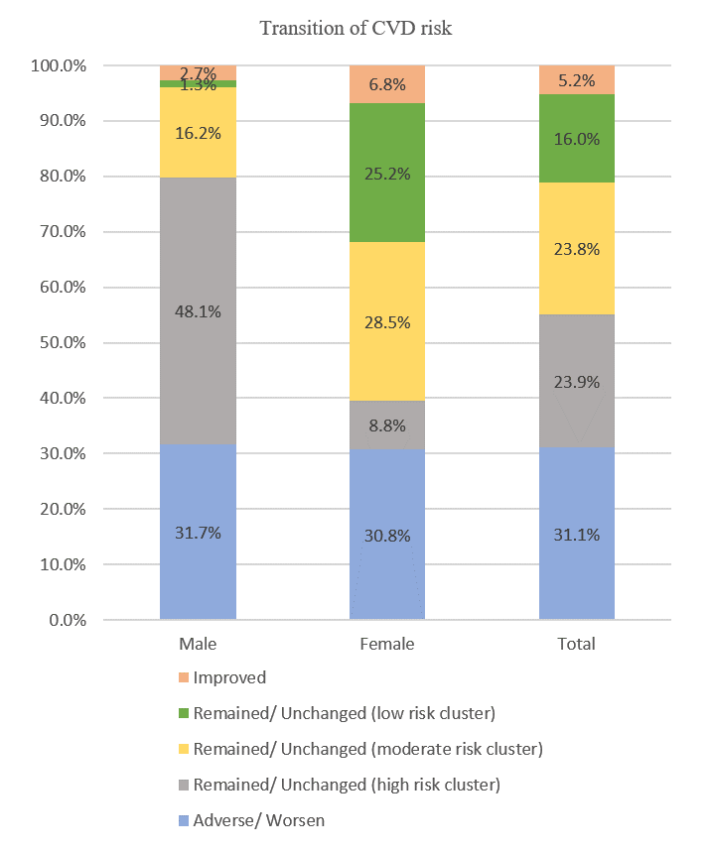
Percentages of predicted cardiovascular disease (CVD) risk trajectories by sex from baseline in 2013 to follow-up in 2018. The distinct trajectory patterns identified were improved; remained unchanged in low, moderate, or high CVD risk clusters; and adverse (worsen) CVD risk trajectories. Notes: improved—the predicted CVD risk had shifted from the high-risk cluster in 2013 to the low-risk cluster in 2018, and adverse (worsen)—the predicted CVD risk had shifted from the low-risk cluster in 2013 to the high-risk cluster in 2018.

Figure 3 present the transition of overall and sex-specified predicted CVD risk trajectories from 2013 to 2018. As can be seen in Figure 3, 33.37% of participants had worsened their CVD risk (from low or moderate in 2013 to high in 2018; Most of the participants remained in the high, moderate, or low CVD risk group at both time points with percentages of 24.38% (n=1609), 22% (n=1452), and 12.35% (n=815), respectively. Only 7.9% (n=521) of the study participants showed improvement in their predicted CVD risks. Sex-stratified analyses revealed that almost half (n=1193, 45.1%) of the male participants remained in the high CVD risk cluster at both time points, 19.4% (n=513) of male participants remained in the moderate-risk group, whereas only 2.08% (n=55) remained in the low-risk category. In contrast, most female participants remained in the moderate (n=939, 23.75%) and low (n=760, 19.22%) CVD risk classification over time. Only 5.16% (n=204) of females had a high CVD risk at both time points. Interestingly, the prevalence of female participants who had improved predicted CVD risk (higher risk in 2013 to lower risk in 2018) was lower than that of males (n=516, 5.16% vs n=317, 11.98%).

### Factors Associated With 10-Year CVD Risk Classification

In the present study, high predicted CVD risk groups from both 2013 and 2018 were predominantly male, of older age (60-69 years), of Malay ethnicity, married, and had a lower SES (completed only up to the primary level of education, did not have a full-time paid employment or self-employment, and earned <RM 3000 (equivalent to US $1000) than the lower CVD risk categories (Table S5 in Multimedia Appendix 1). Frequent eating outside was not significantly different across the CVD risk groups at both time points, although the proportion of participants in the high CVD risk group who did not report having meals outside increased from 37.46% (n=726) in 2013 to 54.78% (n=1600) in 2018. Low physical activity was significantly associated with high CVD risk in 2018 but not in 2013. Regarding mental health, the high CVD risk group was less likely to report mental health conditions (depression, anxiety, or stress).

Table 4 displays the multivariable linear regression models for the associations of demographic, socioeconomic, lifestyle, and mental health factors with changes in CVD risk from baseline to follow-up (CVD risk score at baseline, 2013 – CVD risk score at follow-up, 2018). A negative value indicates an increase in CVD score (worsening), and a positive value indicates a decrease in CVD score (improvement). Chinese and Indian ethnic groups were associated with a decline in CVD risk scores compared to Malays (Chinese: β=.62, 95% CI .39-.84; Indian: β=.33, 95% CI .44-.62). Being unmarried (single, widowed, or other marital status) was associated with an increase in CVD risk scores compared to being married (β=–.52, 95% CI –.77 to –.26). With regard to SES, low monthly income (<RM 1000) was associated with a decrease in CVD risk score (β=.50, 95% CI .25-.76), whereas full-time self-employment and paid employment were associated with an increase in CVD risk score (self-employment: β=–.64, 95% CI –1.00 to –.29; paid employment [full time, part time, and casual job]: β=–.78, 95% CI –1.12 to –.44). The significant associations remained even after further adjustments for behavioral and mental health factors (model 2 and model 3). In model 3, there was an inverse association between severe depressive symptoms and changes in CVD risk score (–1.25, 95% CI –2.44 to –.06; *P*=.04). However, there was no significant evidence for associations between educational levels, lifestyle behaviors (frequency of meals taken outside and physical activity), and 2 other mental health conditions (anxiety and stress) and changes in CVD risk scores. ANOVA tests assessed if adding predictors enhances model fit. A low *P* value suggests that a more complex model (eg, model 3 with added variables) significantly improves fit over a simpler model (eg, model 1 with fewer variables). The ANOVA value indicated that model 3 had the preferred fit, supported by significant ANOVA values that were consistent across all models, even in the most complex models.

**Table 4:** β estimates, 95% CIs, and *P* values of the prospective associations of baseline demographic, socioeconomic, lifestyle, and mental health factors with changes in cardiovascular disease risk score (n=6599)^a^.

Variables		Framingham risk score changes^b^							
		Model 1			Model 2			Model 3	
		β (95% CI)	*P* value	β (95% CI)		*P* value	β (95% CI)		*P* value
ANOVA		2636.4^c^	—^d^	2599.6^c^		—	2658.2^c^		—
**Ethnicity**									
	Aborigine or others	.59 (−.30 to 1.48)	.19	.64 (−.29 to 1.57)		0.18	1.53 (−.21 to 1.69)		.13
	Chinese	.62 (.39 to .84)	<.001	.56 (.34 to .79)		<.001	.56 (.33 to .79)		<.001
	Indian	.33 (.04 to .62)	.03	.29 (−.01 to .58)		0.06	.30 (.00 to .60)		.04
	Malay	1	—	1		—	1		—
**Marital status**									
	Nonmarried	−.52 (−.77 to −.26)	<.001	−.54 (−.80 to −.28)		<.001	−.52 (−.78 to −.26)		<.001
	Married	1	—	1		—	1		—
**Education**									
	Other	.45 (−.17 to 1.06)	.15	.45 (−.17 to 1.07)		.15	.41 (−.21 to 1.03)		.19
	Primary	.19 (−.31 to .69)	.46	.22 (−.28 to .72)		.39	.20 (.30 to .70)		.43
	Secondary	−.27 (−.77 to .22)	.28	−.22 (−.71 to .28)		.40	−.21 (−.71 to .29)		.40
	Tertiary	1	—	1		—	1		—
**Monthly income^b^ (RM)**									
	>1000	.50 (.25 to .76)	<.001	.50 (.24 to .76)		<.001	.50 (.35 to .76)		<.001
	1000-1999	−.04 (−.28 to .19)	.71	−.05 (−.28 to .18)		.68	−.07 (−.30 to .16)		.55
	2000-2999	.12 (−.14 to .39)	.36	.10 (−.17 to .37)		.46	.11 (−.16 to .38)		.43
	≥3000	1	—	1		—	1		—
**Employment**									
	Other	.02 (−.30 to .33)	.92	.02 (−.29 to .34)		.89	.00 (−.32 to .32)		.99
	Self-employed	−.64 (−1.00 to −.29)	<.001	−.66 (−1.01 to −.30)		<.001	−.68 (−1.04 to −.31)		<.001
	Paid employee	−.78 (−1.12 to −.44)	<.001	−.75 (−1.10 to −.40)		<.001	−.73 (−1.08 to −.38)		<.001
	Not working	1	—	1		—	1		—
Frequent outside meals		—	—	−.01 (−.03 to .00)		.12	−.01 (−.03 to .00)		.13
**Physical activity levels**									
	High	—	—	.23 (−.16 to .61)		.24	.23 (−.15 to .62)		.24
	Moderate	—	—	.28 (−.12 to .68)		.17	.27 (−.13 to .67)		.19
	Low	—	—	1		—	1		—
**Depression**									
	Severe	—	—	—		—	−1.25 (−2.44 to −.06)		.04
	Moderate	—	—	—		—	−.10 (−.68 to .48)		.74
	Mild	—	—	—		—	.10 (−.38 to .58)		.68
	No symptom	—	—	—		—	1		—
**Anxiety**									
	Severe	—	—	—		—	−.35 (−1.16 to .46)		.40
	Moderate	—	—	—		—	−.15 (−.57 to .26)		.47
	Mild	—	—	—		—	.07 (−.42 to .55)		.79
	No symptom	—	—	—		—	1		—
**Stress**									
	Severe	—	—	—		—	.63 (−.93 to 2.19)		.43
	Moderate	—	—	—		—	.64 (−.53 to 1.80)		.29
	Mild	—	—	—		—	−.11 (−.87 to .65)		.78
	No symptom	—	—	—		—	1		—

^a^Depicted are β estimates, 95% CIs, and *P* values estimated by multivariable linear regression models.

^b^US $1 was equivalent to RM 3.00 on May 13, 2013, and RM 3.97 on May 17, 2018.

^c^*P*<.001.

^d^Not applicable.

### Sensitivity Analyses

The transition between CVD risk clusters was categorized as either “improved” (the CVD risk scores became better in 2018), “adverse” (the CVD risk worsened in 2018), or “unchanged (high CVD risk)” (being stable at high CVD risk groups) in comparison to being stable in low or moderate-risk groups at both time points. Therefore, we further analyzed multinomial logistic regression models to examine the association between baseline demographic, socioeconomic, lifestyle, and mental health factors and changes in CVD risk groups (no changes or remained low or moderate CVD risk as the reference category; Table S6 in Multimedia Appendix 1).

Among all investigated factors associated with CVD risk trajectories, lower educational levels and frequently having meals from outside were significantly associated with increased odds of worsening CVD risk trajectories. Aborigines and other ethnic groups (compared with Malays), income groups of RM 2000 to RM 2999 and <RM 1000 (compared with >RM 3000), and physical activity were significantly associated with decreased odds of being in adverse CVD risk trajectories.

In addition, lower educational levels and frequently having meals outside were associated with increased odds of remaining in high CVD risk trajectories. Meanwhile, Indian, aborigines, and other ethnicity; being not married; and not being employed for full time (self-employed or others) were associated with decreased odds of remaining in high CVD risk trajectories. However, income levels and physical activity were not associated with unchanged high CVD risk trajectories.

In terms of improvement in CVD risk, income <RM 3000 (compared to higher income levels) and self-employment or other types of employment (compared to not working) were associated with decreased odds of having improved CVD risk score from 2013 to 2018. Other demographic, socioeconomic, lifestyle, and mental health factors were not significantly associated with improved CVD risk trajectories.

Of note, mental health factors were not significantly associated with all CVD risk group trajectories.

## Discussion

### Principal Findings

This study evaluated the changes in CVD risk over a 5-year follow-up period and factors associated with the CVD risk clustering in 6599 multiethnic community-dwelling men and women. Tracking of CVD risk clusters over 5 years of follow-up time revealed that most participants remained in their baseline CVD risk clusters, with 24.38% (n=1609) of the total population remaining in the same high CVD risk profile and 22% (n=1452) in the moderate CVD risk profile, which was dominated by male participants. More alarming, 33.37% (n=2202) of study participants experienced extreme shifts from lower predicted CVD risks in 2013 to higher risks in 2018. This appeared to be reflected by the increased prevalence of all major CVD risk factors over the 5-year follow-up. Of note, our analyses revealed that demographic, SES, depressive symptoms, and dietary habits were important determinants of increasing CVD risk scores and worsening CVD risk trajectories over time.

### Comparison With Prior Work

In this study, we found that most participants (n=3811, 55.37%) had increased CVD risks or remained in the high CVD risk category, which aligns with the rise in all major CVD risk factors from 2013 to 2018. Previous studies in Malaysia confirmed our findings on the distribution of baseline CVD risk categories using the FRS model, with 20% to 23% at high risk, 29% to 38.5% at moderate risk, and 41% to 48% at low risk [26,27]. However, when compared to other studies, including ours, a recent study found a slightly lower prevalence of the high CVD risk category (16.8%) [37]. Despite recent health care service advancements and CVD preventive measures in Malaysia, our analysis confirmed the increasing trend in individual CVD risk factors, as demonstrated by previous nationwide estimates that reported an increase in the prevalence of diabetes and obesity (diabetes: 13.4% in 2015 to 18.3% in 2019; for obesity [BMI≥27.5 kg/m²]: 17.7% in 2015 to 19.7% in 2019), and hypertension remained high at 30% in both years, all of which are comparable to our study (n=2202, 33.37%) [38]. These data have enormous public health implications, suggesting that the increase in major risk factors in Malaysia may place individuals at higher CVD risk, which is supported by other studies from Asia or multiethnic settings showing an upward trend in CVD risk over time. Li et al [39] showed that the increase in the high CVD risk category was primarily due to an increase in the prevalence of hypertension and diabetes. A prospective population-based study from the Multi-Ethnic Study of Atherosclerosis (N=6800) has assessed temporal changes in individual CVD risk factors and found that almost half (45%) of participants remained in the unfavorable CVD risk category [40]. The Multi-Ethnic Study of Atherosclerosis, like ours, also showed an increase in poor blood glucose control over the follow-up period, suggesting more participants’ glucose control deteriorated or became medication dependent later in life, as observed in this investigation. Similar to our findings, the higher predicted CVD risk score may be attributable to the increased prevalence of individual risk factors, such as obesity [41] and smoking [42], translating into a higher prevalence of diabetes mellitus and hypertension [43]. Thus, this study emphasizes the importance of CVD risk factors management, which could be potential candidates for lifestyle or pharmacological interventions explicitly targeted at improving the cardiovascular health of the high-risk groups.

However, the observed increase in mean FRS scores between 2013 and 2018 and the higher prevalence of stable high-risk and worsened CVD-risk categories of this investigation is in contrast with the findings from the United States and Europe [44-46]. First, a US nationwide study using the FRS found a reduction in mean 10-year risk of CVD from 9.2% to 8.7% between 1999 and 2010, among those aged 30 to 74 years, with a larger decline in risk score and proportion of change from high to low risk in men than women [44]. Second, the Tromsø study reported that the mean of the NORRISK 2 CVD risk score decreased and distribution in risk categories moved from higher to lower risk in both sexes and all age groups between the first (2007-2008) and second surveys (2015-2016) [46]. Similarly, a study from England reported a decrease in high risk (QRISK2 score>20%) of 2.4% and 6.8% and medium risk (QRISK2 ≥10%) of 3.2% and 5.3%, in 10-year risk of CVD per decade during 1994 to 2013, for women and men, respectively [45]. Moreover, both increases and decreases in CVD risk score trajectories were associated with CVD risk and disease-free life-years, respectively [8,9], suggesting that higher scores were associated with increased CVD risks, while an improvement in CVD risk scores over a 5-year period was linked to reduced risk. This demonstrates the beneficial effect of reducing modifiable risk factors in the general population, resulting in improved CVD risk factors due to effective preventive strategies. In contrast, a population-based study among 3699 individuals (aged 53 years) from Iran indicated that most low-risk individuals remained low risk and high-risk individuals remained high risk over 10-year follow-up period, confirming our findings on the stability of the CVD risk clusters over 5 years [47]. Despite the favorable increases in high-density lipoprotein and decreased smoking and total cholesterol trajectory patterns, Koohi et al [47] found no evidence of a worsening CVD risk trajectory, which could be explained by the unfavorable increases in fasting glucose.

Of note, previous research demonstrated that traditional cardiometabolic risk factors do not fully account for or explain the excess burden of CVD in the population [13,48]; therefore, assessing other contributing factors of CVD risks is paramount. In this study, differential associations between employment status and changes in CVD risks were observed. First, being employed was associated with a continuous increase in CVD risk score, supporting a previous cross-sectional study, where paid employees experienced the highest prevalence of CVD risk factors compared to the other employment categories [49]. Indeed, employed individuals who are exposed to stress at work, termed as “job strain,” are at an increased risk of coronary heart disease [50,51]. Second, self-employment and others (pensioners, homemakers, and students), which can be linked to both stable and unstable financial situations, were associated with having a high CVD risk at both time points as well as improvement in CVD risk trajectories. Evidence indicates a modest association between job insecurity and incident coronary heart disease, partly attributable to unstable employment status, poorer socioeconomic circumstances, and less favorable risk factor profiles among people with job insecurity [18]. This highlights the potential exposure to job strain in employed individuals and job insecurity in financially unstable types of employment that may influence an individual’s cardiovascular health. In general, having continuous, formal, full-time, and stable jobs provide an income that allows people to meet their basic needs and leads to a better quality of life in adulthood and old age [52,53].

Although the first line of evidence presents conflicting findings with a lower monthly income associated with a decrease in CVD risk score, results from the sensitivity analysis further revealed that lower educational status, used as a proxy of low SES, was associated with adverse CVD risk trajectories, supporting previous evidence on the role of socioeconomic disparities in substantiating CVD risks. Our findings resonate with previous studies among the low-income urban population in Kuala Lumpur, whereby individuals in the lowest income category (<RM 1000) had the highest prevalence of CVD risk factors, and unstable employment was associated with a higher risk of developing CVD [49]. Moreover, epidemiological studies have demonstrated that lower SES is related to risky health behaviors, such as regular smoking and excessive alcohol consumption, that are significant risk factors for the onset of CVD [54]. Semirural residents considered socioeconomically disadvantaged in the LMIC settings have poorer lifestyle behaviors, have a greater tendency to be unable to purchase health-promoting products and services, and experience more negative life events (such as unemployment, marital conflict, and financial hardship), which would lead to negative mental health [55-57]. Consequently, these prolonged stresses may also trigger compulsive behaviors such as overeating, excessive drinking, and tobacco use [58,59].

Regarding lifestyle risk factors, our sensitivity analysis reveals a significant association between diet and adverse CVD risk in a semirural setting. Consistent with previous cross-sectional findings, consuming meals frequently from outside was significantly associated with adverse CVD risk trajectory [31]. The high CVD risk among populations is primarily due to the high salt content of food brought from outside; therefore, health promotion programs focusing on salt reduction should be prioritized. Against expectation, our investigation revealed that physical activity was not significantly associated with changes in CVD risk. Although the protective effects of leisure time physical activity were consistent with a lower CVD risk score in previous studies [60], the lack of association between the risk score and physical activity may have been attributable to the low percentage of participants who are physically active and the homogeneity of activity levels among the study participants from this semirural setting, as demonstrated in a previous study [61].

We additionally found a preliminary association between depressive symptoms and a decline in the CVD score; however, this effect was not observed with CVD risk trajectories as the outcome. One speculation is due to the high prevalence of smoking, which could be associated with lower mental stress levels in the study population. Smoking is highly prevalent in low SES settings in Malaysia and is associated with depression and anxiety among individuals in low-income urban areas [62]. Although longitudinal studies show that smoking cessation (compared with continued smoking) is associated with reduced stress, anxiety, and depression [63], approximately 40% of smokers in England report that they smoke to cope with stress or anxiety [64]. Moreover, according to the stress paradigm, socioeconomic disadvantage is both a source of adversity and a strain on an individual’s coping abilities [65]. Given these circumstances, health-risky behaviors (eg, smoking, overeating, and inactivity) may represent forms of pleasure and relaxation that regulate mood among the population considered disadvantaged. Despite financial instability, maintaining a healthy lifestyle should remain a priority. For instance, even with unstable employment status, a life course free of regular tobacco and alcohol use shows protective effects against CVD in the general population [54]. Therefore, more research on understanding the impact of stress-induced risky behaviors in lower SES groups are warranted.

### Limitations

This prospective study included a large sample of community-dwelling men and women with a high response rate and strict quality assessment. One of the limitations of this prospective study is that direct cause-and-effect relationships between identified factors at baseline and follow-up cannot be discerned. The adapted FRS did not include several other potential CVD risk factors, family history of CVD, and cholesterol levels. Although we have adjusted for a comprehensive set of confounding variables, we cannot exclude that risk factors not included herein may have biased the results. We also acknowledge the potential selection bias in the study due to the exclusion of participants with missing CVD risk factors. Nevertheless, as demonstrated in the dropout analysis, it is expected that older individuals would have higher FRS scores and were also more likely to be excluded from the study. Moreover, several studies have investigated behavioral risk factors in relation to CVD incidence and suggested that adhering to a combination of healthy behaviors (nonsmoking, moderate alcohol intake, physical activity, and fruit and vegetable consumption) was associated with a lower risk of CVD morbidity and mortality [45,66]. However, in our study, we used 2 additional self-reported behavioral factors (frequent eating out and physical activity) in relation to the objective measure used in previous studies. Furthermore, the definition of diabetes would be more accurate if fasting blood samples or HbA_1C_ were assessed. Finally, data on alcohol consumption in Malaysia are underreported due to social and religious aspects.

### Conclusions

Our findings highlight the high prevalence of individuals from a semirural multiethnic setting remaining in high CVD risk clusters and worsening CVD trajectories as they are potentially in the progression of developing CVDs. To our knowledge, this study is the first to examine demographic, socioeconomic, behavioral, and mental health factors associated with changes in CVD risk categories, and our results highlight psychosocial disparities that will perpetuate an unacceptable status quo if left unaddressed.

Both increases and decreases in CVD risk score trajectories were associated with CVD risk and disease-free life-years, respectively. Therefore, interventions targeting an improvement and relatively stable low CVD risk trajectories are favorable, as it indicates a lower risk for the future onset of CVD [67,68]. Recent data from several LMICs in Asia reported that hypertension treatment and control remain challenging in underresourced settings [69]. Therefore, more population-based prevention efforts that focus on CVD risk factors control among populations considered vulnerable should be emphasized. To this end, health care providers should continuously monitor individuals’ CVD risk using updated risk scoring methods and consider any changes in risk factors.

## Appendix

Multimedia Appendix 1 Supplementary material.

## Abbreviations

BP: blood pressure
CVD: cardiovascular disease
FRS: Framingham risk score
HDSS: health and demographic surveillance system
LMIC: low- and middle-income country
MUHREC: Monash University Human Research Ethics Committee
SEACO: South East Asia Community Observatory
SES: socioeconomic status

## Data Availability

The data sets generated during and analyzed during this study are available from the corresponding author on reasonable request.
