# Supplementary material for: Changes in 10-Year Predicted Cardiovascular Disease Risk for a Multiethnic Semirural Population in South East Asia: Prospective Study
Source: JMIR Public Health Surveill. 2024 Sep 26;10:e55261. doi: 10.2196/55261 (PMC11467610; doi:10.2196/55261)
Supplement: Multimedia Appendix 1 [file publichealth_v10i1e55261_app1.docx]

Multimedia Appendix 1: Supplementary Material.

Table S1: Characteristics of included versus excluded study participants.

| Variables | Included (N=6,599) | | Excluded (N=404) | | *P* value |
| --- | --- | --- | --- | --- | --- |
| FRS score mean (± SD) | 11.7 (± 5.5) | | 16.1 (± 4.8) | | <.001 |
| Age groups, n (%) |  | | | |  |
| 35 to 39 | 504 | 7.6 | 23 | 5.7 | <.001 |
| 40 to 49 | 1,549 | 23.5 | 32 | 7.9 |  |
| 50 to 59 | 2,232 | 33.8 | 92 | 22.8 |  |
| 60 to 69 | 1,665 | 25.2 | 105 | 26.0 |  |
| 70 & above | 649 | 9.8 | 152 | 37.6 |  |
| Household income^a^, n (%) |  | | | |  |
| Below RM1000 | 1,371 | 20.8 | 133 | 32.9 | <.001 |
| RM1000 – RM1999 | 1,952 | 29.6 | 107 | 26.5 |  |
| RM2000 – RM2999 | 1,200 | 18.2 | 68 | 16.8 |  |
| RM3000 and above | 2,076 | 31.5 | 96 | 23.8 |  |
| Gender, n (%) |  | | | |  |
| Male | 2,645 | 40.1 | 147 | 36.4 | 0.14 |
| Female | 3,954 | 59.9 | 257 | 63.6 |  |

Notes:

1. Missing values are excluded from the calculations.

**^a^** USD $1 is equivalent to RM3.00 (13^th^ May 2013).

Table S2: Participant demographic and socioeconomic characteristics (N=6,599)

| Variable | 2013 | 2018 |
| --- | --- | --- |
|  |  |  |
| Gender, n (%)  Male  Female | 2,645 (40.1)  3,954 (59.9) | |
| Age in years, mean (±SD) | **55.29 (10.60)** | **60.19 (10.56)** |
| Marital status, n (%) |  |  |
| Married | 5,551 (84.2) | 5,096 (77.2) |
| Never married | 214 (3.2) | 217 (3.3) |
| Separated/Divorced | 115 (1.7) | 158 (2.4) |
| Widow/Widower/Others | 712 (10.8) | 1,128 (17.1) |
| Education, n (%) |  |  |
| Primary | 2,976 (45.7) | 3,034 (46.0) |
| Secondary | 2,898 (44.5) | 2,728 (41.4) |
| Tertiary | 236 (3.6) | 282 (4.3) |
| Others | 405 (6.2) | 548 (8.3) |
| Household income^a^, n (%) |  |  |
| Below RM1000 | 1,371 (20.8) | 1,278 (19.4) |
| RM1000 – RM1999 | 1,952 (29.6) | 1,750 (26.5) |
| RM2000 – RM2999 | 1,200 (18.2) | 1,386 (21.0) |
| RM3000 and above | 2,076 (31.5) | 2,185 (33.1) |
| Occupation, n (%) |  |  |
| Paid employee | 1,577 (23.9) | 1,579 (23.9) |
| Self-employed | 1,246 (18.9) | 850 (12.9) |
| Not working | 724 (11.0) | 1,194 (18.1) |
| Others | 3,052 (46.2) | 2,976 (45.1) |

^a^ USD $1 is equivalent to RM3.00 (13 May 2013) & RM3.97 (17 May 2018) respectively.

**Table S3.** Cardiovascular disease risk factors in 2013 and 2018 among the South East Asia Community Observatory study participants (prevalence: n (%) and mean, SD; N=6599).

|  | 2013, mean (SD) | 2018, mean (SD) |  | *t* test (df) | *P* value^a^ |
| --- | --- | --- | --- | --- | --- |
| Age (years) | 55.3 (10.6) | 60.2 (10.6) |  | –795.8(6598) | <.001 |
| Diastolic blood pressure | 79.0 (10.8) | 77.3 (11.3) |  | 11.5(6598) (*<*.001) | <.001 |
| Systolic blood pressure | 132.8 (19.7) | 134.4 (20.5) |  | –6.3(6598) | <.001 |
| Blood glucose | 8.0 (3.6) | 8.3 (4.1) |  | –4.7(6597) | <.001 |
| BMI | 27.3 (5.1) | 27.6 (5.4) |  | –8.0 (6589) | <.001 |
| Framingham risk score points | 11.7 (5.5) | 14.2 (5.4) |  | –53.3(6598) | <.001 |
| Cardiovascular disease risk (%) | 14.1 (9.8) | 17.8 (9.9) |  | –38.7 (6598) | <.001 |

^a^*P* values for differences between baseline (2013) and follow-up (2018) were calculated using the *χ*^2^ test for categorical variables and the *t* test for continuous variables.

**Table S4.** Sex-stratified cardiovascular disease risk factors in 2013 and 2018 among the South East Asia Community Observatory study participants (prevalence: n (%) and mean, SD; N=6599).

| Variables | Male (N=2645) | | | | Female (N=3954 | | | |
| --- | --- | --- | --- | --- | --- | --- | --- | --- |
|  | 2013, mean (SD) | 2018, mean (SD) | *t* test (df) | (*P* value) | 2013, mean (SD) | 2018, mean (SD) | *t* test(df) | (*P* value) |
| Age (years) | 56.7 (10.7) | 61.6 (10.7) | −508.8 (2644) | <.001 | 54.4 (10.4) | 59.2 (10.4) | –612.0 (3953) | <.001 |
| Diastolic blood pressure | 79.7 (10.8) | 77.5 (11.5) | 8.9 (2644) | <.001 | 78.6 (10.7) | 77.2 (11.1) | 7.5 (3953) | <.001 |
| Systolic blood pressure | 134.0 (18.4) | 134.9 (19.3) | −2.3 (2644) | .02 | 132.0 (20.5) | 134.1 (21.3) | –6.2 (3953) | <.001 |
| Blood glucose | 8.1 (3.3) | 8.3 (4.0) | −4.3 (2644) | <.001 | 8.1 (3.8) | 8.3 (4.1) | –2.6 (3952) | .009 |
| BMI | 26.3 (4.5) | 26.5 (4.8) | −3.6 (2639) | <.001 | 28.0 (5.4) | 28.4 (5.6) | –7.3 (3949) | <.001 |
| Framingham risk score points | 12.7 (4.8) | 15.7 (5.0) | −42.3 (2644) | <.001 | 11.0 (5.8) | 13.1 (5.4) | –34.9 (3953) | <.001 |
| Cardiovascular disease risk (%) | 20.3 (9.0) | 21.9 (9.1) | −10.7 (2644) | <.001 | 9.9 (8.0) | 15.1 (9.5) | –42.6 (3953) | <.001 |

^a^*P* values for differences between baseline (2013) and follow-up (2018) by sex were calculated using the *χ*^2^ test for categorical variables and the *t* test for continuous variables.

**Table S5.** Bivariate analysis of the demographic, socioeconomic, lifestyle, and mental health factors by cardiovascular disease risk categories in 2013 and 2018 among the South East Asia Community Observatory study participants (N=6599)^a^.

| Variables | | | Cardiovascular disease risk categories | | | | | | | | |
| --- | --- | --- | --- | --- | --- | --- | --- | --- | --- | --- | --- |
|  | | | 2013 | | | | 2018 | | | | |
|  | | | Low (n=1820) | | Moderate (n=2814) | High (n=1965) | Low (n=989) | | Moderate (n=2689) | | High (n=2921) |
|  | | | | | | | | | | | |
| **Age groups, chi-square (*df*)** | | | 3165.3 (8) | |  |  | 2699.4 (8) ^c^ | |  | |  |
|  | | 35-39, n (%) | 442 (24.29) | | 61 (2.17) | 1 (0.04) | 4 (0.41) | | 1 (0.04) | | 0 (0.00) |
|  | | 40-49, n (%) | 849 (46.65) | | 615 (21.86) | 85 (4.33) | 604 (61.07) | | 541 (20.12) | | 38 (1.31) |
|  | | 50-59, n (%) | 437 (24.01) | | 1283 (45.59) | 512 (26.06) | 292 (29.52) | | 1055 (39.23) | | 549 (18.79) |
|  | | 60-69, n (%) | 92 (5.05) | | 714 (25.37) | 859 (43.72) | 89 (9.00) | | 848 (31.54) | | 1325 (45.36) |
|  | | ≥70, n (%) | 0 (0.00) | | 141 (5.01) | 508 (25.85) | 0 (0.00) | | 244 (9.07) | | 1009 (34.54) |
| **Sex, chi-square (*df*)** | | | 1553.5 (2) ^c^ | |  |  | 1008.5 (2) ^c^ | |  | |  |
|  | | Male, n (%) | 194 (10.66) | | 1019 (36.21) | 1432 (72.88) | 127 (12.84) | | 826 (30.72) | | 1838 (62.92) |
|  | | Female, n (%) | 1626 (89.34) | | 1795 (63.79) | 533 (27.12) | 862 (87.16) | | 1863 (69.28) | | 1083 (37.08) |
| **Ethnicity, chi-square (*df*)** | | | 44.9 (8) ^c^ | |  |  | 33.5 (8) ^c^ | |  | |  |
|  | Aborigine, n (%) | | 16 (0.88) | | 38 (1.35) | 20 (1.02) | 11 (1.11) | | 43 (1.60) | | 25 (0.86) |
|  | Chinese, n (%) | | 402 (22.09) | | 598 (21.25) | 500 (25.45) | 216 (21.84) | | 591 (21.98) | | 606 (20.75) |
|  | Indian, n (%) | | 252 (13.85) | | 299 (10.63) | 200 (10.18) | 143 (14.46) | | 327 (12.16) | | 282 (9.65) |
|  | Malay, n (%) | | 1116 (61.31) | | 1852 (65.81) | 1234 (62.80) | 601 (60.77) | | 1691 (62.89) | | 1970 (67.44) |
|  | | Others, n (%) | 34 (1.87) | | 27 (0.96) | 11 (0.55) | 18 (1.82) | | 37 (1.37) | | 38 (1.30) |
| **Marital status, chi-square (*df*)** | | | 51.47 (4) ^c^ | |  |  | 67.9 (4) ^c^ | |  | |  |
|  | | Married, n (%) | 1575 (86.78) | | 2330 (82.83) | 1646 (83.81) | 816 (82.51) | | 2086 (77.58) | | 2194 (75.11) |
|  | | Never married, n (%) | 84 (4.63) | | 88 (3.13) | 42 (2.14) | 40 (4.04) | | 117 (4.35) | | 60 (2.06) |
|  | | Others, n (%) | 156 (8.59) | | 395 (14.04) | 276 (14.05) | 133 (13.45) | | 486 (18.07) | | 667 (22.83) |
| **Education, chi-square (*df*)** | | | 612.4 (6) ^c^ |  | |  | 561.4 (6) ^c^ |  | |  | |
|  | | Primary, n (%) | 474 (26.30) | | 1306 (47.18) | 1196 (61.49) | 234 (23.66) | | 1128 (42.00) | | 1672 (57.32) |
|  | | Secondary, n (%) | 1165 (64.65) | | 1194 (43.14) | 539 (27.71) | 648 (65.52) | | 1242 (46.24) | | 838 (28.73) |
|  | | Tertiary, n (%) | 102 (5.66) | | 86 (3.10) | 48 (2.47) | 73 (7.38) | | 125 (4.65) | | 84 (2.88) |
|  | | Other, n (%) | 61 (3.39) | | 182 (6.58) | 162 (8.33) | 34 (3.44) | | 191 (7.11) | | 323 (11.07) |
| **Household income^b^ (RM), chi-square (*df*)** | | | 56.8 (6) ^c^ |  | |  | 25.0 (6) ^c^ |  | |  | |
|  | | <1000, n (%) | 378 (20.77) | | 500 (17.77) | 493 (25.09) | 212 (21.44) | | 548 (20.37) | | 518 (17.74) |
|  | | 1000-1999, n (%) | 496 (27.25) | | 902 (32.05) | 554 (28.19) | 288 (29.12) | | 703 (26.14) | | 759 (25.98) |
|  | | 2000-2999, n (%) | 307 (16.87) | | 531 (18.87) | 362 (18.42) | 212 (21.44) | | 524 (19.49) | | 650 (22.25) |
|  | | ≥3000, n (%) | 639 (35.11) | | 881 (31.31) | 556 (28.30) | 277 (28.00) | | 914 (34.00) | | 994 (34.03) |
| **Employment, chi-square (*df*)** | | | 527.7 (6) ^c^ |  | |  | 414.4 (6) ^c^ |  | |  | |
|  | | Paid employee, n (%) | 500 (27.47) | | 727 (25.84) | 350 (17.81) | 309 (31.24) | | 733 (27.26) | | 537 (18.38) |
|  | | Self-employed, n (%) | 199 (10.93) | | 481 (17.09) | 566 (28.80) | 102 (10.31) | | 356 (13.24) | | 392 (13.42) |
|  | | Not working, n (%) | 96 (5.28) | | 241 (8.56) | 387 (19.70) | 54 (5.47) | | 321 (11.94) | | 819 (28.04) |
|  | | Others, n (%) | 1025 (56.32) | | 1365 (48.51) | 662 (33.69) | 524 (52.98) | | 1279 (47.56) | | 1173 (40.16) |
| **Meals from outside, chi-square (*df*)** | | | $8.0 (6)$ | |  |  | 10.6 (6) | |  | |  |
|  | | 0 meals/week, n (%) | 709 (39.68) | | 1039 (37.43) | 726 (37.46) | 536 (54.20) | | 1456 (54.15) | | 1600 (54.78) |
|  | | 1-5 meals/week, n (%) | 724 (40.51) | | 1155 (41.61) | 774 (39.94) | 298 (30.13) | | 787 (29.27) | | 778 (26.63) |
|  | | 6-10 meals/week, n (%) | 174 (9.74) | | 260 (9.36) | 209 (10.78) | 97 (9.81) | | 293 (10.90) | | 355 (12.15) |
|  | | ≥11 meals/week, n (%) | 180 (10.07) | | 322 (11.60) | 229 (11.82) | 58 (5.86) | | 153 (5.68) | | 188 (6.44) |
| **Physical activity levels, chi-square (*df*)** | | | 5.6 (4) | |  |  | 264.1 (4) ^c^ | |  | |  |
|  | | Low, n (%) | 1624 (89.23) | | 2485 (88.31) | 1721 (87.58) | 155 (15.67) | | 644 (23.95) | | 1119 (38.31) |
|  | | Moderate, n (%) | 102 (5.71) | | 145 (5.15) | 118 (6.01) | 240 (24.27) | | 614 (22.83) | | 656 (22.46) |
|  | | High, n (%) | 94 (5.06) | | 184 (6.54) | 126 (6.41) | 594 (60.06) | | 1431 (53.22) | | 1146 (39.23) |
| **Depression, chi-square (*df*)** | | | 7.7 (2)^e^ |  | |  | 0.03 (2) |  | |  | |
|  | | No symptom, n (%) | 1589 (88.23) | | 2402 (86.34) | 1720 (88.89) | 965 (97.67) | | 2629 (97.77) | | 2854 (97.74) |
|  | | At least mild, n (%) | 212 (11.77) | | 380 (13.66) | 215 (11.11) | 23 (2.33) | | 60 (2.23) | | 66 (2.26) |
| **Anxiety, chi-square (*df*)** | | | 7.4 (2) ^e^ |  | |  | 0.4 (2) |  | |  | |
|  | | No symptom, n (%) | 1525 (84.39) | | 2272 (81.43) | 1628 (83.40) | 946 (95.85) | | 2579 (95.98) | | 2792 (95.65) |
|  | | At least mild, n (%) | 282 (15.61) | | 518 (18.57) | 324 (16.60) | 41 (4.15) | | 108 (4.02) | | 127 (4.35) |
| **Stress, chi-square (*df*)** | | | 9.4 (2) ^d^ |  | |  | 7.2 (2) ^e^ |  | |  | |
|  | | No symptom, n (%) | 1712 (94.90) | | 2658 (95.34) | 1883 (96.81) | 973 (98.58) | | 2671 (99.40) | | 2902 (99.35) |
|  | | At least mild, n (%) | 92 (5.10) | | 130 (4.66) | 62 (3.19) | 14 (1.42) | | 16 (0.60) | | 19 (0.65) |

^a^Differences between baseline (2013) and follow-up (2018) were calculated using the *χ*^2^ test.

^b^US $1 was equivalent to RM3.00 on May 13, 2013, and RM3.97 on May 17, 2018.

^c^ *P*<.001.

^d^ *P*<.01.

^e^ *P*<.05.

**Table S6**: Adjusted odd ratios (95% CI and P-value) from multinomial logistic regression assessing the association between baseline sociodemographic, socioeconomic, lifestyle and psychological factors and changes of predicted CVD risks from baseline in 2013 to follow-up in 2018 of SEACO study participants (N=6,599) (Reference group: no changes (low or moderate remained).

| **Variables** | CVD risk changes ^a^ | | | | | | | | |  |
| --- | --- | --- | --- | --- | --- | --- | --- | --- | --- | --- |
|  | **Model 1** | | | **Model 2** | | | **Model 3** | | |  |
|  | **Improved^b^**  **(N=521)** | **Adverse^c^**  **(N=2,202)** | **No changes**  **(H)^d^**  **(N=2,267)** | **Improved^b^**  **(N=521)** | **Adverse^c^**  **(N=2,202)** | **No changes**  **(H)^d^**  **(N=2,267)** | **Improved ^b^**  **(N=521)** | **Adverse^c^**  **(N=2,202)** | **No changes**  **(H)^d^**  **(N=2,267)** |  |
| **Race** | |  |  |  |  |  |  |  |  |  |
| Aborigine & others | | 0.74  (0.40, 1.36) | 0.46***  (0.30, 0.70) | 0.25***  (0.15, 0.43) | 0.75  (0.40, 1.40) | 0.49**  (0.32, 0.76) | 0.27***  (0.16, 0.46) | 0.78  (0.42, 1.47) | 0.49**  (0.32, 0.76) | 0.29***  (0.17, 0.49) |
| Indian | | 1.25  (0.93, 1.68) | 0.81*  (0.67, 0.98) | 0.76*  (0.61, 0.96) | 1.26  (0.93, 1.71) | 0.83  (0.68, 1.00) | 0.79*  (0.63, 1.00) | 1.28  (0.94, 1.73) | 0.83  (0.68, 1.00) | 0.79*  (0.62, 0.99) |
| Chinese | | 1.36**  (1.08, 1.71) | 0.88  (0.76, 1.03) | 0.97  (0.82, 1.14) | 1.27  (0.99, 1.62) | 0.91  (0.78, 1.06) | 0.96  (0.80, 1.14) | 1.24  (0.97, 1.60) | 0.91  (0.78, 1.07) | 0.94  (0.79, 1.12) |
| Malay | | 1 | 1 | 1 | 1 | 1 | 1 | 1 | 1 | 1 |
| **Marital status** | | | | | | | | | | |
| Non-married | | 0.87  (0.66, 1.16) | 1.15  (0.97, 1.36) | 0.72**  (0.59, 0.87) | 0.88  (0.66, 1.16) | 1.13  (0.95, 1.35) | 0.72**  (0.59, 0.88) | 0.87  (0.66, 1.16) | 1.14  (0.96, 1.35) | 0.72**  (0.59, 0.88) |
| Married | | 1 | 1 | 1 | 1 | 1 | 1 | 1 | 1 | 1 |
| **Education** | |  |  |  |  |  |  |  |  |  |
| Others | | 1.62  (0.87, 3.01) | 2.11***  (1.40, 3.17) | 4.06***  (2.49, 6.62) | 1.82  (0.96, 3.44) | 2.12***  (1.40, 3.19) | 4.16***  (2.55, 6.80) | 1.85  (0.97, 3.53) | 2.12***  (1.41, 3.21) | 4.22***  (2.57, 6.93) |
| Primary | | 1.39  (0.86, 2.26) | 1.50*  (1.09, 2.06) | 3.17***  (2.11, 4.76) | 1.50  (0.91, 2.48) | 1.49*  (1.08, 2.05) | 3.12***  (2.07, 4.69) | 1.57  (0.94, 2.61) | 1.50*  (1.09, 2.06) | 3.24***  (2.14, 4.89) |
| Secondary | | 0.71  (0.44, 1.15) | 1.14  (0.83, 1.55) | 0.96  (0.64, 1.44) | 0.76  (0.46, 1.26) | 1.11  (0.81, 1.52) | 0.94  (0.62, 1.41) | 0.79  (0.48, 1.32) | 1.11  (0.81,1.52) | 0.96  (0.63, 1.45) |
| Tertiary | | 1 | 1 | 1 | 1 | 1 | 1 | 1 | 1 | 1 |
| **Monthly income** | | | | | | | | | | |
| Below RM1000 | | 0.93  (0.72, 1.19) | 0.56***  (0.47, 0.66) | 1.08  (0.89, 1.31) | 0.95  (0.73, 1.22) | 0.57***  (0.48, 0.68) | 1.10  (0.90, 1.33) | 0.96  (0.74, 1.24) | 0.57***  (0.48, 0.68) | 1.09  (0.90, 1.32) |
| RM1000 – RM1999 | | 0.52***  (0.40, 0.68) | 0.87  (0.75, 1.102) | 1.02  (0.86, 1.22) | 0.53***  (0.41, 0.70) | 0.89  (0.77, 1.04) | 1.03  (0.86, 1.23) | 0.55***  (0.42, 0.72) | 0.89  (0.76, 1.03) | 1.03  (0.86, 1.23) |
| RM2000 – RM2999 | | 0.69*  (0.52, 0.92) | 0.80*  (0.67, 0.95) | 0.97  (0.79, 1.19) | 0.69*  (0.52, 0.93) | 0.80*  (0.67, 0.96) | 0.97  (0.79, 1.19) | 0.69*  (0.52, 0.93) | 0.79**  (0.66, 0.94) | 0.95  (0.77, 1.17) |
| RM3000 and above | | 1 | 1 | 1 | 1 | 1 | 1 | 1 | 1 | 1 |
| **Occupation** | |  |  |  |  |  |  |  |  |  |
| Others | | 0.41***  (0.30, 0.58) | 0.97  (0.76, 1.24) | 0.23***  (0.18, 0.29) | 0.43***  (0.31, 0.61) | 0.99  (0.78, 1.26) | 0.24***  (0.19, 0.30) | 0.43***  (0.30, 0.61) | 1.00  (0.78, 1.28) | 0.23***  (0.18, 0.30) |
| Self-employed | | 0.53***  (0.37, 0.76) | 0.86  (0.67, 1.12) | 0.28***  (0.22, 0.36) | 0.54**  (0.38, 0.78) | 0.88  (0.68, 1.14) | 0.27***  (0.21, 0.35) | 0.54**  (0.37, 0.78) | 0.88  (0.68, 1.15) | 0.27***  (0.21, 0.35) |
| Paid employee | | 0.95  (0.65, 1.38) | 0.97  (0.74, 1.28) | 0.822  (0.64, 1.06) | 1.00  (0.69, 1.46) | 1.00  (0.76, 1.33) | 0.84  (0.65, 1.09) | 1.00  (0.68, 1.47) | 1.03  (0.77, 1.37) | 0.82  (0.63, 1.07) |
| Not working | | 1 | 1 | 1 | 1 | 1 | 1 | 1 | 1 | 1 |
| **Frequency of meals taken outside per week** | | | | | | | | | | |
| 11 meals and above | |  |  |  | 1.34  (0.97, 1.87) | 1.31*  (1.06, 1.62) | 1.41**  (1.11, 1.79) | 1.30  (0.93, 1.82) | 1.28*  (1.03, 1.58) | 1.38*  (1.08, 1.76) |
| 6 – 10 meals | |  |  |  | 1.04  (0.73, 1.48) | 1.02  (0.81, 1.27) | 1.27  (0.99, 1.63) | 1.05  (0.74, 1.50) | 0.97  (0.77, 1.22) | 1.23  (0.96, 1.58) |
| 1 – 5 meals | |  |  |  | 0.92  (0.73, 1.15) | 1.03  (0.89, 1.18) | 1.06  (0.91, 1.25) | 0.90  (0.71, 1.13) | 1.01  (0.88, 1.17) | 1.06  (0.90, 1.24) |
| 0 meal | |  |  |  | 1 | 1 | 1 | 1 | 1 | 1 |
| **Level of total physical activity** | | | | | | | | | | |
| High | |  |  |  | 0.99  (0.68, 1.45) | 0.73*  (0.56, 0.96) | 0.85  (0.64, 1.12) | 1.00  (0.68, 1.46) | 0.73*  (0.56, 0.95) | 0.81  (0.61, 1.08) |
| Moderate | |  |  |  | 1.03  (0.69, 1.54) | 0.83  (0.64, 1.09) | 0.94  (0.70, 1.26) | 1.05  (0.70, 1.57) | 0.84  (0.64, 1.10) | 0.91  (0.68, 1.23) |
| Low | |  |  |  | 1 | 1 | 1 | 1 | 1 | 1 |
| **Depression** | |  |  |  |  |  |  |  |  |  |
| Mild to severe | |  |  |  |  |  |  | 0.92  (0.58, 1.45) | 0.87  (0.66, 1.14) | 0.80  (0.59, 1.10) |
| Normal | |  |  |  |  |  |  | 1 | 1 | 1 |
| **Anxiety** | |  |  |  |  |  |  |  |  |  |
| Mild to severe | |  |  |  |  |  |  | 1.08  (0.73, 1.58) | 1.15  (0.91, 1.44) | 1.05  (0.81, 1.36) |
| Normal | |  |  |  |  |  |  | 1 | 1 | 1 |
| **Stress** | |  |  |  |  |  |  |  |  |  |
| Mild to severe | |  |  |  |  |  |  | 0.97  (0.52, 1.78) | 1.21  (0.85, 1.74) | 0.98  (0.63, 1.53) |
| Normal | |  |  |  |  |  |  | 1 | 1 | 1 |
| Goodness of Fit | | 1260.188*** | | | 4163.095** | | | 5903.789*** | | |

Notes: USD $1 is equivalent to RM3.00 (13th May 2013).

^a^Reference category: No changes (L&M) (predicted CVD risk remained low or moderate in both year)

^c^Improved: The predicted CVD risk had become lower/better in year 2018

^d^Adverse: The predicted CVD risk had become higher/worsen in year 2018

^e^No changes (H): The predicted CVD risk remain high in both years

t-test significance: *** P <.001, ** P <.01, *P<.05.

**Supplementary Figure 1: Transition of CVD risk by gender in 2018 (baseline: 2013).**

**(Male, n= 2,645)**

|  |  | 2018 | | | |
| --- | --- | --- | --- | --- | --- |
| **2013** | Risk Cluster | Low | Moderate | High | Total |
|  | Low | 55 | 130 | 9 | 194 |
|  | Moderate | 78 | 513 | 428 | 1,019 |
|  | High | 7 | 232 | 1,193 | 1,432 |
|  | Total | 140 | 875 | 1,630 | 2,645 |

| Colour | Status |
| --- | --- |
|  | Remained/ Unchanged (high risk cluster) |
|  | Remained/ Unchanged (low and moderate risk cluster) |
|  | Adverse/ Worsen |
|  | Improved |

**(Female, n=3,954)**

|  |  | 2018 | | | |
| --- | --- | --- | --- | --- | --- |
| **2013** | Risk Cluster | Low | Moderate | High | Total |
|  | Low | 760 | 760 | 106 | 1,626 |
|  | Moderate | 87 | 939 | 769 | 1,795 |
|  | High | 2 | 115 | 416 | 533 |
|  | Total | 849 | 1,814 | 1,291 | 3,954 |

**(Total, n= 6,599)**

|  |  | 2018 | | | |
| --- | --- | --- | --- | --- | --- |
| **2013** | Risk Cluster | Low | Moderate | High | Total |
|  | Low | 815 | 890 | 115 | 1,820 |
|  | Moderate | 165 | 1,452 | 1,197 | 2,814 |
|  | High | 9 | 347 | 1,609 | 1,965 |
|  | Total | 989 | 2,689 | 2,921 | 6,599 |
